# The Congresses—And after

**Published:** 1908-10-31

**Authors:** 


### The Hospital

A Journal of

The Medical Sciences and Hospital Administration.

New Series. No. 86, Vol. IV. [No. 1158, Vol. XLV.] SATURDAY, OCTOBER 31, 1908.

### The Congresses—and After.

The leisure time of the year—which for prominent and zealous professional men is often no leisure time at all—is now past; during the last few months we of the medical profession have had to interest and instruct us the British Medical Association meeting, the International Tuberculosis Congress, the annual assembly of German scientists and medical men, and the Congress of Surgery at Paris. It is natural, then, in the present "season of mists and mellow fruitfulness," to inquire as to the results of all these announcements and deliberations—as to what sort of a harvest medical research can show in 1908. As yet, of course, no answer is possible beyond a surmise. Nowadays medical investigation has so great a range, both intensive and extensive, that before a verdict can be given on the value of a large conference its printed records must be well studied. These generally read very differently from a verbatim report of the week's deliverances, often raced through in competition with the minute hand of the clock, and in lively anticipation of the president's bell. Every volume of Transactions, or of *Berichte*, or of *Comptes-Rendus*, must contain "communications" which have not hitherto justified their name. To guess from the brief reports at present available, it seems as though this year is not to be a memorable one. Happily we may be certain that it is not a notorious one. No over-confident statements, no premature inferences have been made for lay newspapers to exploit. Indeed, in the matter of popular notice the ecclesiastical assemblies have been much more prominent, a distinction we are far from grudging them. But this negative cause for congratulation, namely, that false hopes have not been raised, is obviously no great thing. Yet the *habitué* of medical congresses knows that oftentimes there is not much more to show, or rather that results and findings rarely correspond to the occasion in magnitude. The reason then must be, either that our medical investigators are not as successful as of yore, or else that they find the lecture-room of a congress hall a somewhat uncongenial locality.

There is something to be said in favour of the first view. Latterly no such fruitful advances have been seen as those brought about by Pasteur, Virchow, and Lister. Tropical medicine may fairly be left out of count in making this comparison; its lately solved problems had been conserved untouched by remoteness from the scientific resources of western civilisation. As soon as Manson's insight gave the hint where to look, only tenacity and organisation were required in order to demonstrate various insects as carriers of infection. When at Hong Kong, in 1894, Kitasato and Yersin engaged in what was practically a race to be the discoverer of the plague bacillus, naturally enough they came in about even, both being highly competent bacteriologists. As has been well said, medicine has now got beyond the stage when a discovery can be made by boiling urine in a test-tube, or examining a slice of liver under the microscope. Without much doubt certain lodes are temporarily worked out; and in other directions the ramifications of further advance are beset with fallacies, and dependent upon slow collective effort. As against this, however, one may instance rontgenology, the causal organism of syphilis, and opsonins, to take one or two prominent recent triumphs. These are all of the first order of importance, it is true; but none of them was the sensation of a medical congress.

Certain of the drawbacks of these gatherings as channels of scientific communication have already been noted. Besides these there is the opportunism which to some extent must needs characterise the papers presented. They must be ready by a certain date; to attract any discussion worth having they must be in close relation to some question of the moment, or to some practical issue; they are read to an audience probably already surfeited with highly technical mental pabulum. The convenience of the author has thus to defer to the convenience of the occasion of publication, whereas with ordinary means of communication the reverse is the case. Those who chance to be at once readers of Mr. Henry James, and lovers of fanciful analogy, may recall this writer's unfavourable comparison, on somewhat similar grounds, of the modern drama with the modern novel. Then the prizes awarded are usually given for essays on set subjects, whereas spontaneity and individual predilection are the very life of the best original work. The establishing of more awards like the Rogers Prize of the University of London, or that offered to general practitioners by the Hunterian Society, in both of which instances some freedom of choice is allowed, might arouse British medicine to greater achievements, while not mischievously fostering the crank and the advertiser. For, indeed, academic correctness has not seldom turned out to be no guarantee of right opinion. To take instances not too recent, it may be recalled that men of very fair standing and special authority wrote dissertations proving that syphilis had nothing to do with tabes. No one, again, can hope to be a contributor to a medical congress unless he enjoys a rather high position in the profession; and a certain number of those holding the requisite rank feel bound to contribute something when the occasion presents itself, almost in order, as it were, to justify their existence. Since only a few even of highly qualified doctors are born students, there necessarily results a considerable amount of mediocre verbiage.

